# A Clinical Audit of Orthopaedic Clinical Documentation of Acute Ankle Fractures: A Quality Improvement Project

**DOI:** 10.7759/cureus.49001

**Published:** 2023-11-18

**Authors:** Mohammed Snober, Qamar Mustafa

**Affiliations:** 1 Trauma and Orthopaedics, Dorset County Hospital NHS Foundation Trust, Dorchester, GBR

**Keywords:** medical education & training, quality improvement projects, ankle fractures, clinical audit system, clinical documentation improvement, surgical practice

## Abstract

Background

Orthopaedic ankle fractures are common injuries that require careful assessment, management, and documentation to ensure optimal patient outcomes. Proper documentation plays a critical role in facilitating communication among healthcare professionals, ensuring accurate diagnosis, treatment planning, and monitoring patient progress. Moreover, it is essential for medico-legal purposes and quality improvement initiatives. This article presents a comprehensive clinical audit aimed at evaluating the quality of orthopaedic ankle fracture documentation within a healthcare setting. The aim of this project was to assess the quality and accuracy of ankle fracture documentation within a single centre against the audit standards set by the British Orthopaedic Association (BOA) and the National Institute for Health and Care Excellence (NICE).

Methods

The study was a closed-loop audit utilising both retrospective and prospective analysis of ankle fracture clerking documentation performed by members of the trauma and orthopaedics team. Two audit cycles were completed in total; the first cycle was carried out in January 2020 where data were collected retrospectively from all orthopaedic admissions of ankle fractures. This was then re-audited against the BOA and NICE guidelines and presented to the local clinical governance meeting. A targeted educational intervention was then implemented with the goal of educating and reinforcing to key team members the documentation standards and the importance of accurate clerking documentation. The second cycle was carried out during July 2020 prospectively. All data were collected and collated with a total of nine data parameters analysed. Patients were included if they were skeletally mature and presented with closed malleolar and syndesmotic ankle injuries. Excluded patients were those who presented with open fractures, pilon fractures, and/or were skeletally immature. Data were then re-presented at the clinical governance meeting.

Results

A total of 23 patients were identified in the initial audit cycle and 22 patients in re-audit. On admission, it was found that 86% of patients presenting with ankle fractures had adequate documentation of their injury mechanism, which subsequently improved to 100% following the intervention. Similarly, there was a 71% improvement in precise documentation of clinical findings of ankle fractures. There was a marked improvement in the consistency of examination findings as well, with over 30% improvement in the rate of documentation for sensation status, skin integrity, circulation, and motor function. Results also revealed a 71% improvement in the documentation rates of vascular examinations where a Doppler ultrasound was used or pulses named in the documentation.

Conclusion

Through a targeted educational scheme focussing on the proposed documentation guidelines, we noted a significant improvement in documentation standards and accuracy of ankle fractures in the trauma and orthopaedic department. With ongoing educational input and reinforcement, team members can be supported to maintain a high level of documentation that meets all available standards, which will ultimately lead to improved patient care.

## Introduction

In the realm of modern healthcare, the paramount importance of quality improvement cannot be overstated. The quest for excellence in patient care is an ongoing journey that healthcare organizations undertake to provide the best possible outcomes for patients. One critical aspect of this endeavour is the meticulous documentation of patient encounters, a practice that ensures that healthcare professionals are equipped with accurate information to make informed decisions and deliver high-quality care. As such, accurate, comprehensive, and relevant documentation is an essential part of patient care [1].

Ankle fractures constitute a common presentation to emergency departments nationally, making their accurate diagnosis and management a critical aspect of orthopaedic care. The National Institute for Health and Care Excellence (NICE) and the British Orthopaedic Association (BOA) have established clinical guidelines and standards that serve as a benchmark for excellence in the treatment of ankle fractures [2,3]. These guidelines encompass various aspects of patient care, including assessment, diagnosis, treatment, and follow-up. One fundamental component of adhering to these standards is the systematic and thorough documentation of patient cases, which not only aids in immediate clinical decision-making but also plays a pivotal role in post-treatment evaluation and research. Documentation of salient features as outlined in the guidance, including assessment of circulation, motor function, and sensation, is important to ensure timely decision-making for these injuries.

Despite the vital importance emphasised by these guidelines, accurate and comprehensive documentation may not always be regarded as a priority in an emergency situation, and there is concern that documentation of the trauma patient and the person with non-complex fractures, in particular, is not always optimal [3,4]. This can lead to a myriad of challenges, including diagnostic errors, incomplete patient records, and delayed or inappropriate treatment. The consequences of such shortcomings are not only detrimental to patient outcomes but also place healthcare providers at risk of potential litigation.

It has been noted that orthopaedic admissions do not always reference all of these mandatory findings [5]. Therefore, the purpose of this clinical audit project is to ensure relevant documentation standards, as set out by NICE and BOA, are being met. By comparing the standards of a single centre to these standards, we aim to identify key areas of potential quality improvement, aiming to improve documentation standards of acute ankle fractures. We also endeavoured to set up a comprehensive education initiative to ensure widespread awareness regarding the importance of detailed and relevant documentation.

## Materials and methods

This study was conducted in a single centre, in the surgical care group of trauma and orthopaedics. The approval for the audit was granted by the Clinical Audit Approval Group in 2020, at University Hospitals Dorset, Poole General Hospital (reference #4935). Data in the first audit cycle were collected retrospectively through January 2020, and the second cycle included data from July 2020. Admission documentation for patients presenting with ankle fractures was scrutinised, and documentation standards were checked for compliance with audit standards, taken from the BOA Standards for Trauma and Orthopaedics (BOAST) (Table 1) and NICE (NG 37) standards of practice.

**Table 1:** The British Orthopaedic Association Standards for Trauma – the management of ankle fractures Adapted from [2].

Standards for practice
The mechanism of injury and clinical findings, including skin integrity, assessment of circulation, and sensation, should be precisely documented at presentation.
Comorbidities that might influence treatment choice and outcome should be documented. These might include pre-existing mobility impairment, diabetes mellitus, peripheral neuropathy, peripheral vascular disease, osteoporosis, renal disease, smoking, and alcohol abuse.
Reduction and splinting should be performed urgently for clinically deformed ankles. Radiographs should be obtained before reduction unless this will cause an unacceptable delay.
Radiographs should be centred on the ankle and should include a true lateral and a mortise view.
Additional radiographs of the whole leg are required when clinical examination suggests a more proximal fracture of the fibula (Maisonneuve injury). Separate radiographs of the foot and knee should be obtained if clinically indicated. CT imaging may be helpful in defining fracture configuration in more complex patterns particularly where the posterior malleolus is involved.
Following reduction, the neurovascular examination must be repeated and documented.
Adequate reduction must be confirmed by a review of repeat radiographs and documented before transfer from ED.
Fractures considered stable should be treated with analgesia and splinting, and patients allowed to bear weight as tolerated. Further follow-up may not be necessary.
In fracture patterns where stability is uncertain, patients should be reviewed within two weeks with further radiographs (weight bearing if possible) to confirm the position remains acceptable.
Early fixation (on the day or day after injury) is recommended in the majority of patients under 60 years old when the ankle mortise is unstable. The use of external fixation may be rarely indicated in the presence of gross instability associated with soft tissue compromise.
In patients over 60 years old, close contact casts are an option if reduction can be maintained.
Surgery should aim to achieve reduction and stabilisation of the ankle mortise. The syndesmosis should then be assessed and stabilised if unstable. Intraoperative radiographs should be obtained to confirm reduction.
Most patients should be allowed to bear weight as tolerated in a splint or cast unless there are specific concerns regarding the stability of the fixation or contraindications, such as peripheral neuropathy or particular concerns about the status of the soft tissues.
After surgery, patients should be followed up in a fracture clinic within six weeks of surgery to detect complications and confirm maintenance of reduction on radiographs.
Thromboprophylaxis risk assessment should follow agreed local protocols
All patients should receive information regarding expected functional recovery and rehabilitation, including advice about returning to normal activities such as work and driving. A mechanism should be in place for patients to self-refer to the fracture service if progress is not as anticipated.

Patients were included in the study if they presented with closed malleolar and syndesmotic ankle injuries and were skeletally mature. The exclusion criteria omitted any pilon fractures, open ankle fractures, and ankle fractures in skeletally immature patients, as well as patients who lacked capacity/had reduced cognitive function. This was to allow consistency in the complexity of injuries and fracture patterns that were being analysed and reviewed.

The presence and quality of documentation in orthopaedic clerking were analysed for eight different data parameters. These were formulated using the aforementioned guidelines with the intention of including all parameters that would create precise and comprehensive clerking documentation in line with national standards. These parameters were as follows: (i) mechanism of injury; (ii) skin integrity at presentation; (iii) vascular/circulation status; (iv) vascular/circulation examination, including capillary refill/perfusion status, the warmth of the limb, and examination of named pulses or Doppler ultrasound; (v) neurological examination at presentation, including sensory and motor examinations; (vi) sensory neurological section of the examination, including fine touch sensation, proprioception, pinprick/pain sensation, and documents dermatomal/named peripheral nerve distribution; (vii) motor function section of the neurological exam, including the use of the Medical Research Council (MRC) Scale for Muscle Strength; (viii) neurovascular examination following reduction; (ix) overall documentation of clinical findings at presentation (i.e., the inclusion of all examination findings).

Data from the first audit cycle were collected and analysed. The results were subsequently presented to the surgical care group with the intention of developing an intervention that would lead to an improved and consistent quality of ankle fracture examination documentation. Possible interventions were discussed with numerous members of the orthopaedic multidisciplinary team with an emphasis on finding a sustainable and effective intervention that would allow for the easy reinforcement of the importance of clear documentation.

A targeted educational intervention was implemented with the intention of educating key stakeholders (clerking doctors from the orthopaedic team) about the documentation standards and reinforcing these standards. A teaching programme surrounding patient documentation was set up at local induction meetings with further teaching sessions held at trauma meetings. These sessions focused on patient safety and medico-legal requirements of accurate documentation. Trainees who had attended teaching were then invited to submit case-based discussion reports to consolidate their learning and reinforce the principles taught.

Further data were then recollected, analysed, and re-presented to the surgical care group. There was an in-depth discussion and analysis of common pitfalls that were consistently being found in orthopaedic clerking and the possible causes behind these pitfalls. Following on from this, with minor changes being made, the intervention was made permanent as part of training and education in the department, with regularly scheduled teaching sessions, particularly during induction periods for new doctors starting in the department. An emphasis was also placed on the use of work-based assessment to reinforce the importance of these injuries and the documentation standards associated with them.

## Results

Following the application of inclusion/exclusion criteria, the audit identified 23 patients who had been admitted to the orthopaedic department. All were closed malleolar fractures in skeletally mature patients who had been admitted to an orthopaedic ward, following presentation to the emergency department.

On admission, most patients (86%) had a documented mechanism of injury and some written documentation of clinical findings. Just 17% of all admitted patients had precise documentation of their examination. “Precise” was defined as documentation of sensory, motor, and vascular findings using a dermatome chart, MRC scale, and named pulses. In patients initially seen by an orthopaedic junior doctor, only 54% of admissions documented skin integrity, circulation, or sensation before reduction of the fracture with plaster. Once reduction had taken place, only 56% of admissions had a repeated examination.

Where a neurovascular assessment had been done, only 48% of admissions used the MRC scale to assess power or examined sensation using a dermatome chart. Of admissions, 17% documented named pulses.

Once the orthopaedic team had a presentation on the expected standards of documentation and were reminded of these standards, there was a marked improvement in documentation standards. The re-audit cycle initially identified 22 patients, and following the application of exclusion criteria, 17 patients were identified and included in the audit.

Of the patients admitted, 100% had a documented mechanism of injury. There was a 71% improvement in the precise documentation of overall clinical findings on examination. In the re-audit, there was 100% documentation found of skin integrity and 88% of all clerking clearly documented the circulatory status and sensation on presentation. There was a noticeable improvement in the rates of neurovascular examinations after the reduction of the fracture, improving from 56% to 88%. Similarly, once a neurovascular examination had been performed, there was evidence of motor function assessment using the MRC scale, sensation assessment using a dermatome chart, and documentation of named pulses in 88% of admissions, a 71% improvement for the first cycle. All results can be found in Table 2.

**Table 2:** Analysis of documentation of ankle fracture admissions, with targets according to sources, baseline analysis, analysis following educational intervention, and percentage changes BOAST (ankle): British Orthopaedic Association Standards for Trauma and Orthopaedics, the management of ankle fractures; NICE NG37: National Institute for Health and Care Excellence, fractures (complex): assessment and management (NG37); MRC scale: Medical Research Council Scale for Muscle Strength.

Criteria	Target	Target risk	Source	Actual (first cycle)	Actual (following intervention	% change
The mechanism of injury should be precisely documented at presentation	100	80	BOAST (ankle)	86	100	14
Skin integrity should be precisely documented at presentation	100	80	BOAST (ankle)	54	100	46
Vascular/circulation status should be documented at presentation	100	80	BOAST (ankle)	54	88	34
Vascular/circulation examination includes capillary refill/perfusion status, warmth of the limb, examination of named pulses, or Doppler ultrasound	100	80	NICE NG 37	17	88	71
Neurological examination should be documented at presentation	100	80	BOAST (ankle)	54	88	34
The sensory neurological section of examination includes fine touch sensation, proprioception, and pinprick/pain sensation and documents dermatomal/named peripheral nerve distribution	100	80	NICE NG 37	48	88	40
The motor function section of the neurological exam includes the use of the Medical Research Council Scale for Muscle Strength (MRC scale)	100	80	NICE NG 37	48	88	40
Following reduction, the neurovascular examination must be repeated and documented	100	80	BOAST (ankle)	56	88	32
Overall documentation of clinical findings at presentation	100	80	BOAST (ankle)	17	88	71

## Discussion

The exact impact of incomplete documentation has not been extensively reported in the literature. However, the initial clerking and documentation of ankle fractures is important as it helps clinicians establish a baseline neurovascular status and guide treatment decisions. Neurovascular impairment can be an indication for urgent surgery, and a specific neurological injury can provide an indication of injury pattern and severity [6]. Serial examinations assist in monitoring for neurovascular improvement or compromise and, in patients who later go on to have operations, admission (pre-operative) neurovascular status helps differentiate iatrogenic injuries from those sustained from the mechanism of injury [7]. Additionally, medical guidelines are progressively being utilised to outline standards of care in the medico-legal setting, and medical documentation may be scrutinised in courts of law [8,9]. The BOA mandates precise documentation of clinical findings and therefore this represents a medico-legal issue. Studies also suggest that incongruency between pre-operative findings and documentation resulted in adverse events [10].

This audit has identified that documentation standards of ankle fractures at our centre do not meet national standards. The main issue identified was a failure to completely document neurovascular findings during admission. There are several reasons for these findings revealed through semi-structured interviews of stakeholders.

Firstly, distal neurovascular intact (DNVI) was frequently used as a substitute for documentation of sensory, motor, and vascular findings in lieu of a dermatome chart, MRC scale, or named pulses. We considered shorthand notations to be equivalent to no examination performed. This is because, although they imply an examination was done, they do not provide any details as to how thorough the examination was. It should be emphasised that the lack of complete documentation does not necessarily indicate a lack of complete examination. However, a lack of detailed documentation could lead to questions regarding the thoroughness of the examination should a change in neurovascular status occur. This consensus has been documented in similar studies that audit documentation of orthopaedic admissions [7].

Secondly, staff may have relied on the emergency department clerking when seeing these patients, particularly where reduction may have already taken place. Thirdly, where reduction had already taken place, the presence of a plaster cast was thought to impair a thorough neurovascular examination. Fourthly, all patient findings were documented in generic admission proformas, which do have some prompts to document particular parts of the examination. Introducing a proforma has been shown to improve adherence to documentation standards in a variety of medical settings [9-12]. However, habituation to these prompts can develop, and may also help explain further reasons for the results of this audit.

Based on the national standards, it was clear that documentation standards for ankle fractures were not being met. To rectify this, doctors were educated on and reminded of the duty to document clinical findings pre and post-reduction using validated tools, including dermatome chart, MRC scale, and named pulse check.

The results demonstrate that a targeted educational intervention delivered to key stakeholders can increase the quality of admission documentation recorded. Mechanism of injury and skin integrity was recorded in all cases. Furthermore, clinical findings were precisely documented in the majority of cases, including the use of the MRC scale, dermatome charts, and named pulses. This compares to only 17% of patients during the baseline audit. Only two patients had an incomplete documentation record where the shorthand DNVI continued to be used. As we have previously seen, shorthand notations should be avoided because although they imply an examination was done, they do not provide any details as to how thorough the examination was and can often lead to medical errors and misunderstanding [13].

The results demonstrate that we are meeting national standards for the documentation of ankle fractures. Targeted education is a low-cost, low-resource intervention that is easily deliverable and sustainable, as shown in previous studies [14,15]. To sustain this change, we have identified induction as an important transitory period to deliver this intervention, as part of departmental induction. If this intervention was not successful or proved to not yield sustainable improvements, other suggested interventions included the introduction or amendment of the existing clerking proforma to specifically include sections to document the previously mentioned examination parameters.

In this study, we implemented the Plan-Do-Study-Act (PDSA) cycles, which outline four steps that can be used to test the effectiveness of an intervention. These include planning, trying, observing the results, and acting on what is learned.

Limitations

There are a few limitations that were identified in this study. Firstly, it is a single-centre study, and the findings of the study may not necessarily be applicable or replicated in other larger centres. Furthermore, the retrospective nature of the study may potentially introduce some selection bias, given the lack of study blinding and external review, as well as the inability to control who was performing the examinations (i.e., the study does not reflect on everyone in the department).

## Conclusions

The results suggest that documentation of ankle fractures is improved through a targeted education intervention. We would advise that this teaching be delivered during the induction of new orthopaedic doctors to ensure the change is maintained and that documentation standards are maintained. This intervention can be further applied to other common injuries/presentations. This study has highlighted the immense importance of adequate documentation in a trauma and orthopaedic setting, and how by comprehensively and clearly documenting examinations we can ensure relevant and effective care for our patients. With detailed documentation, departments can integrate safe and effective handover of patient care, ensure reliable communication between team members and even allow for easy access to patient history in the future. It has also emphasised the effectiveness of simple cost-effective measures such as educational interventions.
